# Cerebral carbon dioxide embolism after kidney cancer laparoscopic surgery with full neurological recovery: A case report

**DOI:** 10.1097/MD.0000000000020986

**Published:** 2020-07-02

**Authors:** Yuan Li, Enlong Zhang, Huishu Yuan

**Affiliations:** a Department of Radiology, Peking University Third Hospital, Haidian District; b Department of Radiology, Peking University International Hospital, Life Park Road No.1 Life Science Park of Zhong Guancun, Chang Ping District, Beijing, China.

**Keywords:** cerebral carbon dioxide embolism, laparoscopic surgery, neurological recovery, treatment options

## Abstract

**Rationale:**

Cerebral carbon dioxide embolism (CCDE) is a rare cause of stroke and is a recognized life-threatening complication.CCDE may result from direct intravascular CO_2_ insufflation during surgery. Due to the lack of typical clinical manifestations, the disease is often missed or mistaken for another condition. The clinical signs and symptoms depend on the speed and volume of embolized gas entering the blood and the patient's condition. In particular, patent foramen ovale has been found to be associated, in rare cases, with the intraoperative entry of gas into the arterial system.

**Patient concerns:**

In this report, we present the case of a 35-year-old woman with kidney cancer who underwent laparoscopic right partial nephrectomy.

**Diagnosis:**

After the laparoscopic surgery, the patient was initially diagnosed with acute cerebral infarction.

**Interventions:**

The patient was treated according to the standard method for treatment of acute cerebrovascular disease.

**Outcomes:**

Three days after the laparoscopic procedure, the patient gained consciousness, and she was discharged without any neurologic sequelae on postoperative day 12.

**Lessons subsections as per style:**

Due to the low incidence and sudden occurrence of CCDE, there is a strong likelihood of missed diagnosis or misdiagnosis, and it is; therefore, important to be aware of the risk. The findings from this report would be highly useful as a reference to clinicians in the future.

## 1 Introduction

Laparoscopic surgery is a common technique in abdominal surgery, and its application in clinical practice is on the rise. In laparoscopic procedures, gas insufflation is usually used for accurate surgical visualization and manipulation. Carbon dioxide (CO_2_) is the most commonly used gas for insufflation because it is colorless, inexpensive, and nonflammable.^[1]^ Further, it has higher blood solubility than air, and is; therefore, associated with a reduced risk of complications in case of venous embolism. Although laparoscopic surgery with CO_2_ insufflation is generally a beneficial procedure, it does have the disadvantage of potentially causing complications such as subcutaneous emphysema, hypercapnia, respiratory acidosis, cerebral edema, and CO_2_ embolism.^[2,3]^ Clinically significant CO_2_ embolism is rare and unnoticeable, but it is potentially fatal.^[4]^ The clinical presentation of CO_2_ embolism ranges from asymptomatic to neurologic injury, cardiovascular collapse or even death, and is dependent on the speed and volume of embolized gas entering the blood and the condition of the patient. CO_2_ embolism is typically venous and may also cause pulmonary embolism and acute myocardial infarction. More rarely, the gas is able to cross from venous to arterial circulation, and this is referred to as a “paradoxical embolus.”^[5]^ In paradoxical CO_2_ embolism, CO_2_ from the right heart flows into the left heart through the intracardiac or extracardiac shunt, and patients with patent foramen ovale are likely to develop cerebral carbon dioxide embolism (CCDE), which is a rare, but serious neurological deficit with fatal consequences.^[6]^ Published articles have reported that most patients with CCDE exhibit a transitional stage of delayed recovery from general anesthesia, temporary hemiplegia, and disturbance of consciousness.^[7]^ Here, we present a well-documented case of a patient with patent foramen ovale who had a stroke associated with CCDE that developed after laparoscopic surgery for kidney cancer. The patient was later discharged as she did not exhibit any neurologic sequelae.

## 2 Case presentation

**Figure 1:**
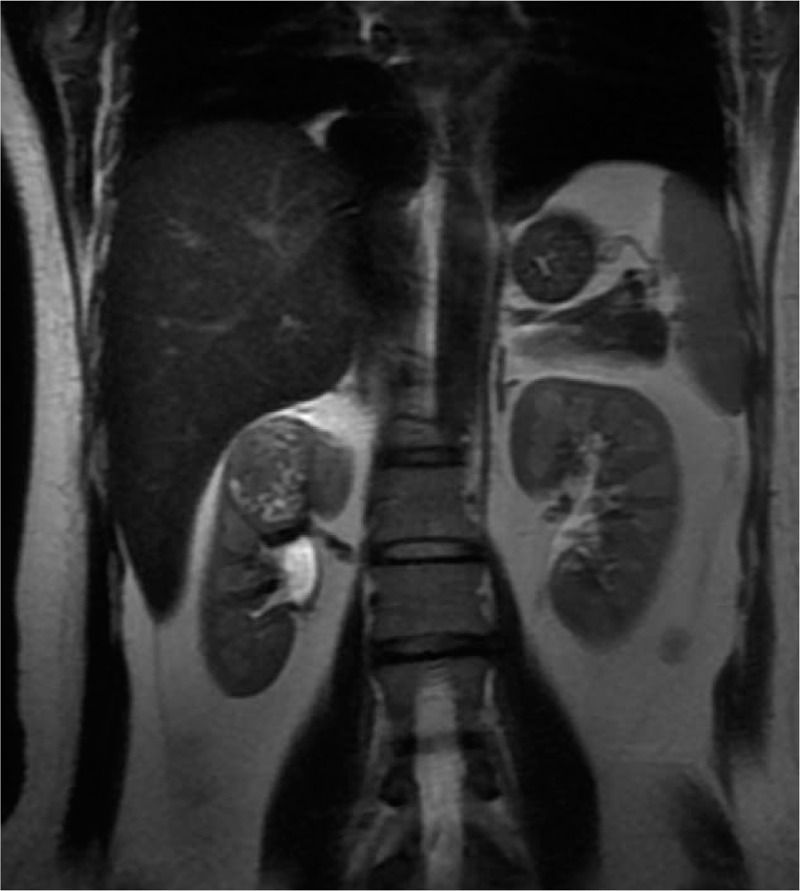
MRI demonstrating a lesion consistent with a primary renal neoplasm. MRI = magnetic resonance imaging.

A 35-year-old female patient (height, 165 cm; weight, 62 kg) presented to our hospital with pain in the right back for more than 1 month. On physical examination, her abdomen appeared soft and nontender, with no palpable masses. Abdominal computed tomography (CT) and magnetic resonance imaging (MRI) revealed a 4.2 × 3.2 × 2.6 cm solid mass in the upper pole of the right kidney (Fig. 1). Renal cell carcinoma was suspected, and the patient was scheduled for a laparoscopic right partial nephrectomy. According to her medical history, she had undergone post-laparoscopic cholecystectomy and surgery for ectopic pregnancy, and was allergic to penicillin. She did not have a family history of renal cell carcinoma or genetic abnormalities.

In preparation for the procedure, general anesthesia was induced and maintained according to the standard protocol. The preoperative heart rate was 88 beats/min; systolic blood pressure (BP), 138/68 mm Hg; and oxygen saturation, 99% to 100%. The patient was placed in the left lateral decubitus position. CO_2_ insufflation was used to establish pneumoperitoneum and maintain the intra-abdominal pressure at 12 mm Hg. Extraperitoneal fat from the surface of the lateral vertebral fascia was removed, and the perirenal fascia was opened along the leading edge of the psoas major. The peripheral lymphatic vessels of the renal pedicle were cut off with an ultrasonic knife, and the renal artery was detached from the dorsal side of the hilum. The kidney fat capsule was opened and attached to the surface of the kidney to dissociate the kidney. Titanium clamps were used to trap small peripheral blood vessels and bleeding points. Laparoscopic arteriotomy forceps were used to block the renal artery. The tumor and some kidney tissue located 0.5 to 1.0 cm away from the tumor edge were removed. The tumor was removed through the expanded camera port incision and placed in an organ bag. The renal pelvis and parenchyma were joined by continuous suture. The renal artery was opened, and any oozing blood was wiped off the wound surface. The bleeding area was pressed down with a hemostatic gauze. The procedure time and warm ischemia time were 186 minutes and 25 minutes, respectively. The estimated blood loss volume during the procedure was 50 mL, and the patient did not require blood transfusion. No vein or artery was injured, and no complications occurred during the surgery. The patient remained hemodynamically stable.

After surgery, the patient was moved to the anesthesia recovery room, and her vital signs were found to be stable after extubation (body temperature, 37.1°C; heart rate, 86/min; BP, 112/63 mm Hg; breathing rate, 20 breathes/min; and oxygen saturation, 92%–95%). The patient was breathing spontaneously, but was still deeply sedated and vomiting frequently. She was also responsive to orbital pressure and pain stimulation. Her clinical symptoms included left hemiparesis, positive left-sided Babinski sign, suspicious positive left-sided Hoffman sign, and increased right facial muscle tension. The left and right pupils were of the same size (about 4 mm in diameter), and the patient was positive for the doll's eye test. In both eyes, the stare was fixed to the top left area of the field of vision. Wrinkling and teeth motion on the right side of the face were observed on pain stimulation. Pain stimulation in the right limb induced some activity, but pain stimulation in the left upper limb and left lower limb resulted in little and no obvious activity, respectively.

**Figure 2:**
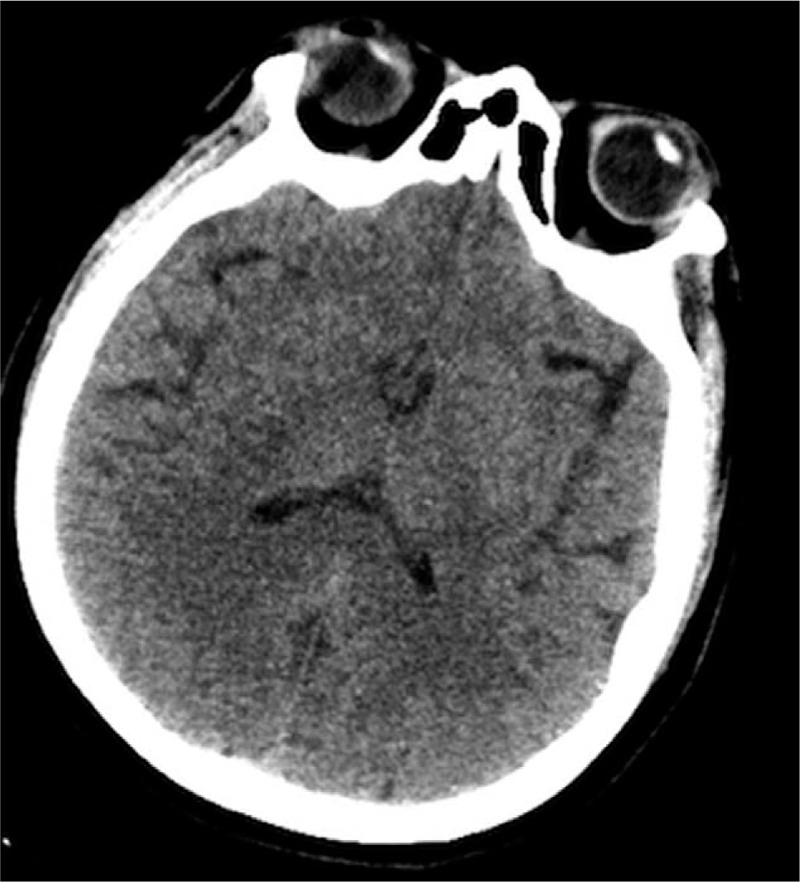
Cerebral CT scan taken 3 h after the surgical procedure showing no obvious abnormalities. CT = computed tomography.

**Figure 3:**
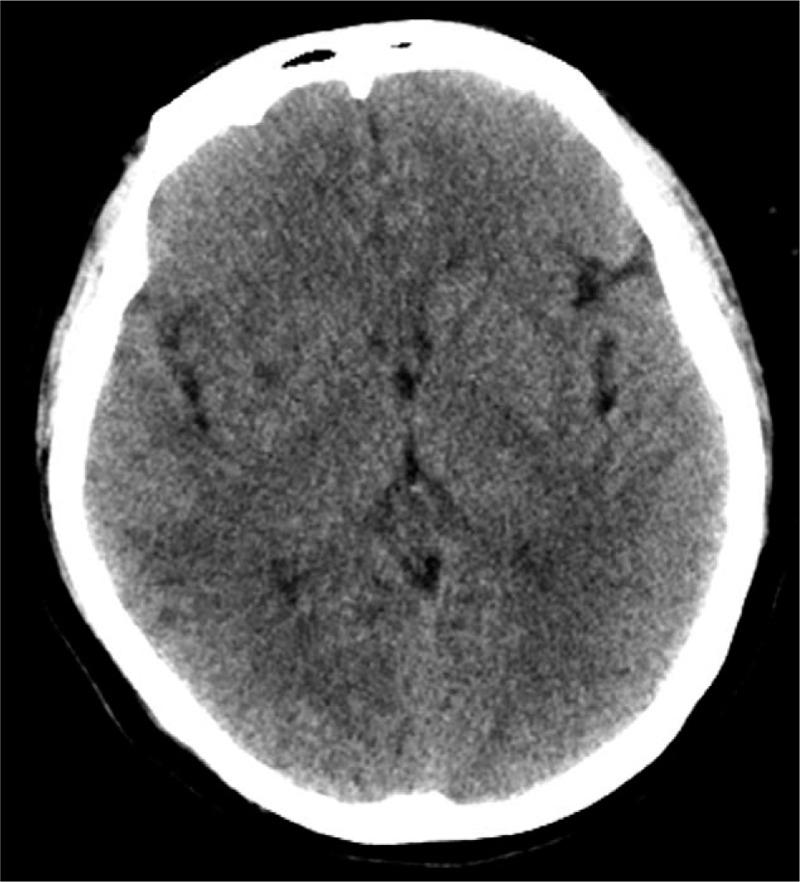
Follow-up CT scan taken 2 d later showing a hypodense lesion in the right hemisphere. CT = computed tomography.

**Figure 4:**
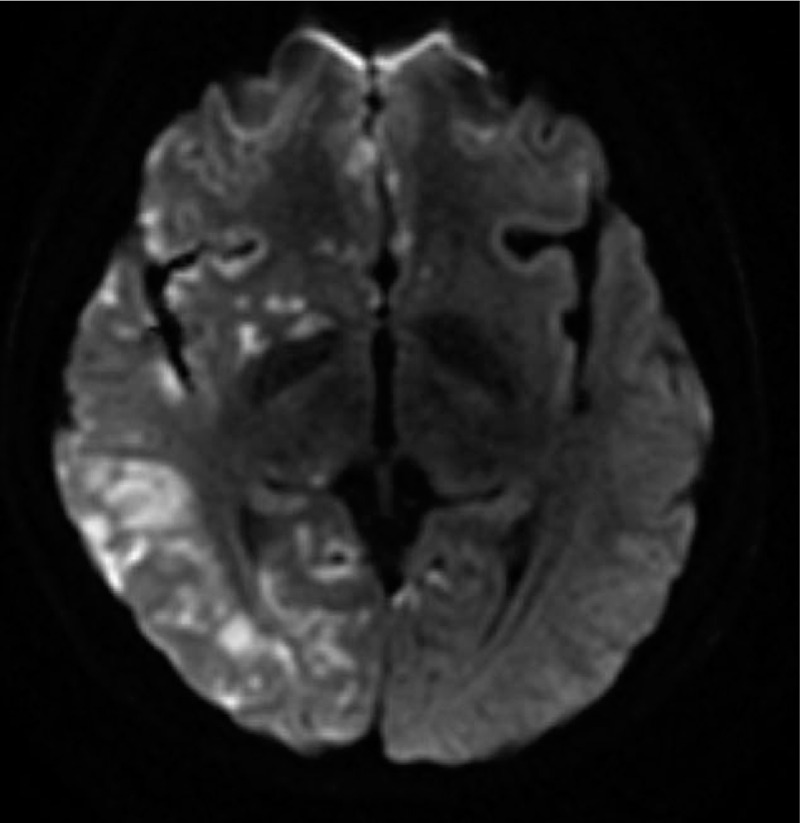
Brain MRI showing paradoxical CO_2_ embolism. The diffusion-weighted imaging (DWI) image revealed multiple cerebral infarction, predominantly on the right side of the cerebral cortex on day 5 after the procedure. MRI = magnetic resonance imaging.

An urgent consultation was arranged with the department of neurology, in order to examine the patient for cerebral infarction and cerebral hemorrhage. An emergency CT scan (taken approximately 3 hours after surgery) showed no bleeding foci or occupying lesions (Fig. 2). This was indicative of cerebral infarction, but not intracerebral hemorrhage, so the latter was excluded. The patient received oxygen ventilation via a mask at a rate of 2 L/min, and was moved to the intensive care unit and connected to a mechanical ventilator. In order to assess for the presence of acute cerebrovascular diseases, emergency brain MRI and cerebrovascular angiography were recommended. The patient was administered midazolam, butorphanol, norepinephrine, and vinpocetine. However, no obvious abnormalities were observed in cerebral blood vessels of the head and neck in the angiography findings. The patient had no previous history of hypertension, diabetes, hyperlipidemia, or abnormality in a local echocardiograph. Plavix and aspirin were prescribed, and treatment for maintaining BP was administered by the neurologists at the department. A repeat CT scan taken 2 days later showed that there was a hypodense lesion in the right hemisphere, but there was no sign of cerebrovascular gas (Fig. 3). Mannitol was administered for intracranial dehydration; edaravone, to eliminate free radicals present in the brain; and dexamethasone, to relieve edema. Apart from symptomatic treatment, plavix and aspirin were administered as antiplatelet therapy. On day 4, the patient regained consciousness but had slow reaction times. On day 5 after the surgery, brain MRI showed multiple cerebral infarction located predominantly on the right side of the cerebral cortex (Fig. 4). Contrast-enhanced echocardiography of the right heart was performed to determine if she had right-to-left systemic shunt in the lung or heart. The result was indicative of patent foramen ovale. Based on the results of brain CT, MRI, cerebrovascular angiography, and contrast echocardiography of the right heart, the patient was eventually diagnosed with CCDE. On day 9, the patient had generally recovered and was discharged with completely normal neurological functions on day 12.

## 3 Discussion and conclusion

The high blood solubility of CO_2_ in plasma can reduce the risk of CO_2_ embolization during laparoscopic surgery via rapid absorption and elimination of CO_2_ through the lungs. However, if a large amount of CO_2_ enters into the vascular system, it can lead to venous CO_2_ embolism, which is rarely seen in humans.^[6]^ The incidence rate of gas embolism during laparoscopic surgery is approximately 0.15%, and the mortality rate can be as high as 30%.^[8]^ CO_2_ embolism has been reported in several laparoscopic procedures, including laparoscopic liver resection, laparoscopic cholecystectomy, laparoscopic appendectomy, and gynecological laparoscopy.^[9]^ The presence of a gas in venous circulation may obstruct pulmonary circulation, and clinically significant CO_2_ embolism can be asymptomatic or even present with cardiac symptoms, including cardiovascular collapse and neurological deficits, and even death.^[9]^ In patients with patent foramen ovale, CO_2_ embolism may take the form of a very rare but fatal paradoxical gas embolism that leads to cerebral gas embolism.

In the present case report, a 35-year-old female patient with kidney cancer who underwent laparoscopic right partial nephrectomy. Although the surgical procedure was successfully completed with perioperative vein or artery injury, cerebral infarction induced by a gas embolism still occurred. To our knowledge, this is the first report of cerebral paradoxical gas embolism with complete neurological recovery after laparoscopic nephrectomy.

During laparoscopic surgery, it is important to be aware of the possibility of CO_2_ embolism and the potential risk factors, including venous bleeding, high ventilatory pressure, patent foramen ovale, and patient positioning. Prompt and accurate recognition and management can limit the clinical impact of such events. The symptoms of gas embolism include systemic hypotension, tachypnea, dyspnea, cyanosis, tachycardia or bradycardia, arrhythmia, and asystole. Paradoxical embolism may also be associated with altered mental status, focal neurological deficits, and loss of consciousness. In our case, a right-to-left shunt caused paradoxical gas embolism when CO_2_ entered through the vein. No obvious vascular injury was observed during the operation (probably because the patient was young), and no obvious bubbles were found in the brain CT scan. This indicates that the embolism was caused by a very small volume of CO_2_ gas. Further, the patient remained hemodynamically stable during the surgery, and this might explain her quick recovery.

We searched PubMed for previously reported cases of complications caused by CO_2_ embolism associated with laparoscopic radical nephrectomy or partial nephrectomy. The following keywords were used: “carbon dioxide embolism,” “nephrectomy,” and “laparoscopy,” as shown in Table 1.^[10–13]^ The findings of the identified studies indicate that CO_2_ embolism is extremely rare in cases of laparoscopic nephrectomy. We also researched the incidence of CCDE during laparoscopic procedures by searching PubMed with the keywords “cerebral gas embolism,” “carbon dioxide,” and “laparoscopy,” and have summarized the patients’ backgrounds, treatments administered, and outcomes in Table 2.^[7,14–20]^ Overall, the findings indicate that clinically significant CO_2_ embolism may be fatal.

**Table 1:**
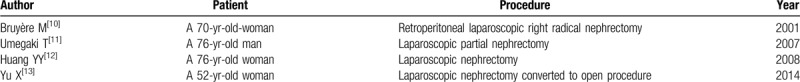
Previous reports on cases of CO_2_ embolism as a complication of laparoscopic radical nephrectomy or partial nephrectomy.

**Table 2:**
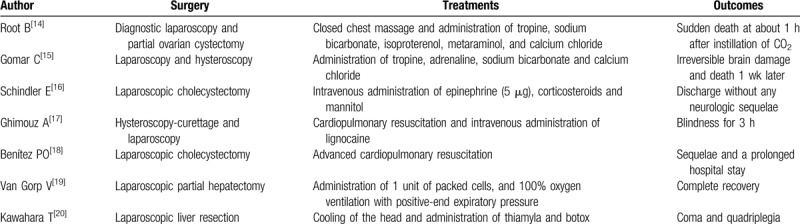
Previous reports on the incidence of cerebral carbon dioxide embolism (CCDE) during laparoscopic procedures.

Preoperative medical evaluation should be conducted to understand the cardiopulmonary risks of a procedure and anticipate possible complications. When gas embolism is suspected, the pneumoperitoneum must be released and the patient must be placed in the Trendelenburg position. Further, if a gas embolism is suspected at any point of time, the surgeon should be notified immediately and insufflation should be discontinued. In order to reduce ventilation-perfusion mismatch and improve hypoxemia, CO_2_ should be washed out with 100% oxygen ventilation.^[21]^ In addition to symptomatic treatment, prevention of cerebral edema, and hyperbaric oxygen therapy, the amount of gas entering the brain could be reduced by keeping the head in a lower position.^[22]^ For patients with severe cardiovascular collapse, supportive treatment with fluid, vasopressors, and cardiopulmonary bypass may be necessary. Currently, there is little literature on standard treatment for cerebral CO_2_ embolism, so the prevention of this condition is very important.

Due to the low incidence and sudden occurrence of CCDE, the possibility of missed diagnosis or misdiagnosis exists. Therefore, it is important to be aware of the risk of this condition, and the findings from this report might prove useful to clinicians in the future.

## Acknowledgment

The authors would like to thank the patient's family for giving consent.

## Author contributions

YL and ELZ designed the idea and wrote the manuscript. YL and ELZ collected the data. HSY edited the manuscript. All authors read and approved the manuscript.
